# The Treatment of Typhoid Fever by Intestinal Antiseptics

**Published:** 1892-10

**Authors:** 


### THE TREATMENT OF TYPHOID FEVER BY INTESTINAL ANTISEPTICS.

This method of treating typhoid fever, already in vogue for sometime, has received great encouragement from the recent paper in the *British Medical Journal* by Dr. Richard Caton, of the Royal Infirmary, Liverpool.

Dr. Caton analyzes forty-four cases, one-half of which he treated by the expectant method; the other half were treated by different antiseptics, as salicylate of soda, naphthalin, creasote, alpha-naphthol, etc., with the purpose of securing a non-septic intestinal canal. Of the twenty-two treated by the expectant method, four died; of the same number treated by the antiseptic plan, none died. In other respects, all the cases received the same treatment. Hyperpyrexia, when it occurred, was treated by tepid baths, or cold sponging in the bed, with the administration of 5-grain doses of quinine. The salicylate of soda is both nauseating and depressing and not so desirable as the other antiseptics named. The author has seen no bad results from creasote, naphthalin or alpha-naphthol. By the expectant method the average duration of fever was 37.9 days; with the antiseptic method it was 25.3 days. Those treated expectantly remained 52 days in hospital; those treated by intestinal antisepsis remained 46 days. As regards relapses, of the 16 who recovered under the expectant plan, the average days of relapse were 9; of the 22 treated antiseptically, the average days of relapse were 1.8.

Dr. Wolff, of Philadelphia, has treated 100 cases with naphthalin. The average febrile period was 24.4 days, and the mortality 2 per cent. The naphthalin is administered in the form of a pill, and 6 or 8 pills, each containing 3 or 4 grains, are given daily.

The author is much impressed by the apparently good effects of the intestinal antiseptic treatment. It is obviously a rational method. The use of antiseptics has lessened the danger arising from external injury to the body; so our experiments now encourage us to believe that, internally applied, they may diminish the mortality from enteric fever.

